# The Impacts of Regulations and Financial Development on the Operations of Supply Chains with Greenhouse Gas Emissions

**DOI:** 10.3390/ijerph15020378

**Published:** 2018-02-22

**Authors:** Zhuang Xiao, Yixiang Tian, Zheng Yuan

**Affiliations:** 1 School of Management and Economics, University of Electronic Science and Technology of China, Chengdu 610054, China; xiaozhuang@cqwu.edu.cn (Z.X.); yuan3931666@126.com (Z.Y.); 2 School of Management and Economics, Chongqing University of Arts and Sciences, Yongchuan 402160, China

**Keywords:** greenhouse gas (GHG), GHG emission regulation, financial development, supply chain management

## Abstract

To establish a micro foundation to understand the impacts of greenhouse gas (GHG) emission regulations and financial development levels on firms’ GHG emissions, we build a two-stage dynamic game model to incorporate GHG emission regulations (in terms of an emission tax) and financial development (represented by the corresponding financing cost) into a two-echelon supply chain. With the subgame perfect equilibrium, we identify the conditions to determine whether an emission regulatory policy and/or financial development can affect GHG emissions in the supply chain. We also reveal the impacts of the strictness of GHG emission regulation, the financial development level, and the unit GHG emission rate on the operations of the supply chain and the corresponding profitability implications. Managerial insights are also discussed.

## 1. Introduction

In recent years, climate change and its implications for the human race have emerged as one of the most important themes in debates about the relationship between global environmental protection and economic performance. An intuitive suggestion is that governments and/or non-governmental organizations (NGOs) should take action to regulate greenhouse gas (GHG)–emitting firms. Such regulations are expected to make firms reduce their GHG emissions. However, empirical observations do not consistently support this expectation.

The inconsistency shown in empirical results can be explained according to two theoretical clues. The conventional view [1,2,3] believes that regulation increases the cost of GHG emissions that accompany a firm’s production, then lowers its production volume and GHG emissions, and finally leads to a negative correlation between environmental and economic performance. However, the Porter hypothesis [4,5,6] argues that regulation increases a firm’s incentive to invest in improving its GHG emission abatement technology, which gives the firm a competitive advantage in the future. Thus the Porter hypothesis indicates both a short-run negative correlation and a long-run positive correlation between environmental and economic performance.

From a practical viewpoint, this empirical inconsistency, together with the corresponding theoretical reasoning, may lead to difficulties for both policy-makers and managers in evaluating the impacts of regulatory policy alternatives and/or choosing appropriate decisions to deal with the interactions between firms’ operations and regulations. Especially when choosing a regulatory policy, a government or an NGO is mainly concerned with whether and under what conditions such a policy is effective in terms of reducing GHG emissions. On the other hand, faced with a possible change in regulatory policy, managers of firms hope to know how regulation affects their operations and the corresponding environmental and economic performance. These questions call for a more careful strategy-based understanding of how firms respond to regulatory policies [7,8,9].

Further, supply chains become a prominent mode to organize production, distribution, and consumption. Due to the existence of interfirm interactions in a supply chain, it is more difficult to analyze the responses of supply chain members to regulatory policies. Moreover, in the setting of a supply chain, resources are usually distributed unevenly among its members [10,11], which implies the existence of some resource-constrained members and the need to relax such constraints by financing from financial markets. Thus the level of financial development can significantly affect the responses of supply chain members to regulatory policies.

Recognizing the interfirm interactions and the uneven distribution of resources, we examine how firms in a two-echelon supply chain strategically respond to government (or NGO) regulation and explore the corresponding implications of environmental (GHG abatement) and economic performance. More specifically, the supply chain consists of an upstream supplier and a downstream retailer. The production of the supplier is accompanied by some GHG emissions, while the downstream retailer is subject to a working capital constraint. The supplier sells the product in question to the retailer via a wholesale price contract, and the retailer resells the product to consumers in the final market. Furthermore, we assume that the government (or NGO) uses a GHG emission tax, a kind of market-based environmental regulation, to regulate the supplier’s GHG emissions. Finally, we assume that the retailer can borrow from an external bank to relax its working capital constraint (whenever needed), and the interest rate depends on the level of financial development (as indicated in [12,13], a higher level of financial development usually leads to lower financing cost). Introducing the retailer’s working capital constraint allows us, on the one hand, to explore the responses of the supplier’s production and the retailer’s borrowing induced by the regulatory policy, and thus identifies the conditions under which a regulatory policy can effectively (or ineffectively) work to decrease the supplier’s GHG emissions. Here, as will see, whether the retailer borrows to relax its working capital constraint is critical in determining the effectiveness of a regulatory policy. On the other hand, it also helps us to establish a theoretical explanation for the empirical observation [14,15,16,17] that there is an inverted U-shaped relationship between GHG emissions and financial development. Indeed, this inverted U-shaped relationship also depends on the retailer’s borrowing decision.

We solve the subgame perfect equilibrium of the supply chain model by backward induction. By analyzing the equilibrium, we contribute to the literature with policy implications in the following three aspects. First, we incorporate GHG emissions regulations and financial development into a uniform framework of supply chain operations. This complements the literature that independently discusses the impact of either regulation [18,19] or financial development [20,21] on GHG emissions. Second, our results identify the conditions under which GHG emission regulation (in terms of an emission tax) and financial development (represented by the corresponding interest rate) effectively affect firms’ GHG emissions and explore how they do so. This helps to provide a theoretical foundation to respond to the current debate on the effectiveness of regulatory policies and the impact of financial development. For example, the inverted U-shaped relationship [14,15,16,17] may be an outcome of different regulatory policies under different financial development levels: the positive (negative) part corresponds to the settings where the effect of financial development dominates (is dominated by) the effect of a strengthened regulatory policy and/or an improved GHG abatement technology. Third, our results imply that financial development may have a significant impact on the effectiveness of a regulatory policy to achieve its GHG emission reduction goal, since a higher level of financial development can incentivize GHG emitters (the supplier, in our case) to emit more. Thus, a GHG emission regulatory policy should be conditional on the financial development level.

The rest of this paper is organized as follows. Section 2 reviews the related literature. Section 3 presents a game model to describe the supply chain operations. Section 4 provides the subgame perfect equilibrium. Section 5 gives the comparative statics with discussions of managerial insights. Concluding remarks are provided in Section 6.

## 2. Literature Review

Our paper is mainly related to three streams of literature.

As for the first stream of literature, authors empirically investigate how emission regulations affect firms’ environmental or economic performance (through improving their emission abatement technologies and/or reducing their production). Chakrabarti and Mitra [22] found that in response to environmental regulations, by adopting pollution-control technologies, firms in relatively small-scale industries in India could increase environmental and social benefits. With data from the Indian cement industry from 2000 to 2004, Mandal [23] demonstrated that environmental regulations significantly improved the industry’s energy efficiency and firms’ financial performance. Sueyoshi and Goto [24] pointed out that big enterprises can effectively manage environmental regulations to improve operational and environmental performance and finally increase their financial performance. Popp [25] explored environmental regulatory pressure triggering firms’ responses by environment-friendly technological innovation. These studies all reflect that firms positively respond to environmental regulations with better environmental and economic performance, implying the effectiveness of regulations to decrease emissions. However, another group of empirical findings tended to suspect such effectiveness. From the perspective of performance, environmental performance and financial performance were found to be positively correlated [26,27,28] or negatively correlated [29,30], or even have no correlation [31,32]. Faced with empirical debates on the effectiveness of environmental regulations, our paper tries to theoretically identify conditions under which an emission tax effectively (or ineffectively) induces the emitting firm in a supply chain (the upstream supplier) with a working capital–constrained downstream retailer to decrease its GHG emissions by reducing its production.

The second stream studies the effects of GHG emission regulations on supply chain operations. Du et al. [33] showed that in a cap-and-trade system, the systemwide and manufacturer’s profits increase with the emission cap, while the permit supplier’s decreases, implying room for coordination. Tseng and Hung [34] proposed a strategic decision-making model to evaluate a supply chain network’s CO_2_ emissions and operational costs under different scenarios and showed that the social cost rate of CO_2_ emissions weakens them in the supply chain network. Sundarakani et al. [35] demonstrated that CO_2_ emissions across stages in a supply chain can influence the design phase of supply chains. Palak et al. [36] explored substantial impacts of different carbon regulatory mechanisms (including carbon cap, carbon tax, carbon cap and trade, and carbon offset) on transportation and inventory replenishment decisions. Zakeri et al. [37] highlighted the role of a carbon trading mechanism in improving a (centralized) supply chain performance. From the perspective of supply chain design, the findings in Chaabane et al. [38], Elhedhli and Merrick [39], and Jin et al. [40] demonstrated the important and different roles of different CO_2_ emission regulations in the design of a supply chain. These authors in general highlighted that CO_2_ emission regulations have a significant impact on the design and operations of supply chains. However, since most of their models were too complex to get closed-form solutions, the theoretical implications and the managerial insights could not be very clearly explained in a logically consistent manner. Our simplified model (but commonly used in the supply chain management literature) allows us to get the closed-form subgame perfect equilibrium and thus complements the literature in this regard, by clearly identifying the conditions to determine whether a GHG emission regulation actually affects the operations of a supply chain and exploring how it works. Then the managerial insights naturally follow.

The third stream examines the role of external financing in relaxing capital constraints in supply chains. Buzacott and Zhang [41] incorporated asset-based financing into production decisions and highlighted the importance of joint consideration of production and financing decisions for start-up firms. Katehakis et al. [42] demonstrated the optimal operational financial policy of an inventory system that admits both interest-bearing loans and interest-earning deposits. Jin and Luo [43] incorporated insurance policies into the well-known news vendor model and examined how the capital level, the bank’s risk aversion, and the insurer’s loading factor affect optimal inventory and insurance decisions. Lekkakos and Serrano [44] studied the impact of reverse factoring schemes on small and medium enterprises’ operational decisions and performance. Yan et al. [45] built a bilevel Stackelberg game model where the bank, as a leader, finances both the supplier and the retailer in a supply chain, and reported an all-win result relative to where, in a no-financing case, financing benefits the bank, the supplier, and the retailer. The results of Srinivasa et al. [46] also showed that a joint decision mode to finance both the supplier and the retailer dominates an independent decision mode to finance these two supply chain members. Kouvelis and Zhao [47] analyzed the channel choice problem between internal trade credit financing and external bank financing. We extend the models in this stream of literature that just capture financing needs by introducing GHG emission to the production in a supply chain. Thus, with an additional interpretation of the financing cost as the level of financial development, we can examine the impacts of both financial development and regulatory policies on the operations of the supply chain and provide corresponding managerial insights.

## 3. The Model

In this section, we build a game-theoretic model to describe the operations of a two-echelon supply chain under a government’s or an NGO’s environmental regulations. The supply chain consists of an upstream supplier and a downstream retailer. The supplier and the retailer conduct transactions over a product with a wholesale price contract. The wholesale price is denoted by w(>0). The retailer resells it to consumers in the final product market. The final market demand function for the product is p=a−bq, where a>0, b>0, and p and q represent the retail price and the quantity demanded by the market, respectively.

The supplier’s production of one unit of the product is accompanied by β(>0) units of GHG emissions. GHG emissions are subject to the government’s or the NGO’s GHG emission tax regulation: each unit of emission is taxed at a rate of γ(>0). Thus, if the supplier produces q units of the product, the regulation-induced cost is c(q)=βγq, which implies that the marginal regulation-induced cost is βγ. Moreover, the supplier may also incur a constant unit production cost $c_{0}$. However, for notational briefness, we follow [48,49] and normalize $c_{0}$ to be zero (i.e., $c_{0}=0$).

The retailer has a certain amount of working capital K>0 and uses fixed working capital to pay the supplier to purchase the product. Thus the retailer may be constrained by the fixed working capital when the amount to pay exceeds K. We assume that the retailer can borrow from an external bank at an interest rate $r_{d}$ (and then the financing cost is $Lr_{d}$ if the amount L is borrowed) to relax its working capital constraint. Since an interest rate is usually viewed as a typical indicator of the cost to finance in the financial market, we use $r_{d}$ to model the extent to which an economy’s financial system is developed: a better-developed financial system attracts more participants (including banks) to compete against one another, and the heightened competitiveness leads to a lower interest rate. Further, as financial development helps to decrease firms’ financing costs [12,13], we use a lower $r_{d}$ to represent a higher level of financial development.

Finally, we assume that βγ<w<p<a, where the first inequality implies that the supplier is willing to produce and sell the product to the retailer, the second implies a positive retail margin to ensure the retailer’s participation, and the third ensures that the sold quantity in the final market is positive.

With the above assumptions, the supplier’s and the retailer’s profit functions can be respectively written as

$$\pi_{s}(w)=(w-\beta \gamma )q(w)$$

and

$$\pi_{r}(q,L)=(a-bq-w)q-Lr_{d}$$

where q(w) is the retailer’s optimal order quantity at the given wholesale price w.

The decision sequence is as follows. In stage 1, the supplier decides the wholesale price w. In stage 2, the retailer chooses the ordering quantity q and the financing amount L from the bank.

## 4. The Equilibrium

We solve the subgame perfect equilibrium by backward induction. In stage 2, given the wholesale price w, the retailer chooses q and L to maximize its profit:

(1) $$\begin{array}{l} \underset{q,L}{\max}\;\pi_{r}(q,L)=(a-bq-w)q-Lr_{d}\\ s.t.\;wq\leq K+L\\ \;L\geq 0 \end{array}$$

Lemma 1 summarizes the retailer’s optimal reaction (with the order quantity q and the financing amount L) to the supplier’s wholesale price decisions. The proofs of all formally stated lemmas and propositions, except Proposition 1, which is a straightforward result of Lemmas 1 and 2 according backward induction, can be found in the Appendix A.

**Lemma** **1.** *In stage 2, given the wholesale price* w*, the retailer’s reaction functions are*

(2) $$q(w)=\left\{ \begin{array}{l} \frac{a-w}{2b},\;\mathrm{if}\;w\geq w_{2}\;\mathrm{or}\;w\leq w_{1}\\ \frac{a-(1+r_{d})w}{2b},\;\mathrm{if}\;w_{3}\leq w\leq w_{4}\\ \frac{K}{w},\;\mathrm{otherwise} \end{array}\right.$$

(3) $$L(w)=\left\{ \begin{array}{l} \frac{[a-(1+r_{d})w]w}{2b}-K,\;\mathrm{if}\;w_{3}\leq w\leq w_{4}\\ 0,\;\mathrm{otherwise} \end{array}\right.$$

*where*

$$w_{1}=\frac{a-\sqrt{a^{2}-8bK}}{2},\;w_{2}=\frac{a+\sqrt{a^{2}-8bK}}{2}$$

$$w_{3}=\frac{a-\sqrt{a^{2}-8bK(1+r_{d}})}{2(1+r_{d})},\;w_{4}=\frac{a+\sqrt{a^{2}-8bK(1+r_{d}})}{2(1+r_{d})}$$

*and* $w_{1}\leq w_{3}\leq w_{4}\leq w_{2}$.

Lemma 1 shows that the retailer borrows a positive amount from the bank only when the supplier’s wholesale price is at some moderate level ($w\in [w_{3},w_{4}]$). This result is quite intuitive. For high enough or low enough wholesale prices ($w\geq w_{2}$ or $w\leq w_{1}$), the retailer’s working capital is enough to cover the spending when buying products from the supplier. As the supplier’s wholesale price goes up to a level higher than $w_{1}$ for a low enough one or falls below $w_{2}$ from a high enough one, the retailer’s working capital becomes binding. In this case, as w increases from $w_{1}$ to $w_{2}$, the marginal value of financing ($\lambda_{1}$) first increases and then decreases after $w_{2}$ exceeds $4bK/a\in (w_{1},w_{2})$. This implies that for $w\in [w_{3},w_{4}]\subset (w_{1},w_{2})$, the marginal value of financing is higher than the marginal financing cost $r_{d}$. Therefore, the retailer responds with a positive financing amount to the supplier’s wholesale price $w\in [w_{3},w_{4}]$. On the other hand, when $w\in (w_{1},w_{3})\;\mathrm{or}\;(w_{4},w_{2})$, the marginal benefit is dominated by the marginal cost, implying that the retailer does not have any incentive to borrow from the bank, even though its operations are constrained by the fixed amount of working capital.

Another implication of Lemma 1 is that financial development has a substantial impact on the retailer’s response to the supplier’s wholesale price decisions. More specifically, not only does an increase in the level of financial development (a decrease in $r_{d}$) enlarge the financing interval $\left[w_{3},w_{4}\right]$, but it also raises the retailer’s demand for the supplier’s product when the supplier’s wholesale price falls within $\left[w_{3},w_{4}\right]$. However, the retailer’s order decision is independent of the level of financial development if $w\notin [w_{3},w_{4}]$. In this case, financial development does not have any impact on either the retailer’s decision or the operations of the supply chain. Thus, how financial development affects the operations of a supply chain also depends on the supplier’s choice of wholesale price. Indeed, as will be immediately shown in Lemma 2 below, the supplier would choose wholesale prices to suppress the retailer’s incentive to finance, even if there is such a set of wholesale prices that allows the retailer to positively finance its working capital constraint.

We now go to the supplier’s decision. In stage 1, with the retailer’s reaction functions, the supplier’s profit function can be rewritten as

(4) $$\pi_{s}(w)=\left\{ \begin{array}{l} \frac{\left(w-\beta \gamma )(a-w\right)}{2b},\;\mathrm{if}\;w\geq w_{2}\;\mathrm{or}\;w\leq w_{1}\\ \frac{\left(w-\beta \gamma )[a-(1+r_{d})w\right]}{2b},\;\mathrm{if}\;w_{3}\leq w\leq w_{4}\\ \frac{K(w-\beta \gamma )}{w},\;\mathrm{otherwise} \end{array}\right.$$

With this profit function, we can derive the supplier’s optimal wholesale price decision, which is summarized in Lemma 2.

**Lemma** **2.** *In stage 1, the supplier’s optimal wholesale price is*

(5) $$w^{\#}=\left\{ \begin{array}{l} \frac{a+\beta \gamma }{2},\;\mathrm{if}\;\sqrt{a^{2}-8bK}\leq \beta \gamma \\ \frac{a+(1+r_{d})\beta \gamma }{2(1+r_{d})},\;\mathrm{if}\;r_{d}\leq r_{d}^{\#},(1+r_{d})\beta \gamma <\sqrt{a^{2}-8bK(1+r_{d})}\;\mathrm{and}\;\sqrt{a^{2}-8bK}>\beta \gamma \\ \frac{a+\sqrt{a^{2}-8bK}}{2},\;\mathrm{otherwise} \end{array}\right.$$

*where* $r_{d}^{\#}$ *is the positive solution to*

(6) $$\frac{{\left[a-(1+r_{d})\beta \gamma \right]}^{2}}{8b(1+r_{d})}=K\left(1-\frac{2\beta \gamma }{a+\sqrt{a^{2}-8bK}}\right)$$

**Figure A2:**
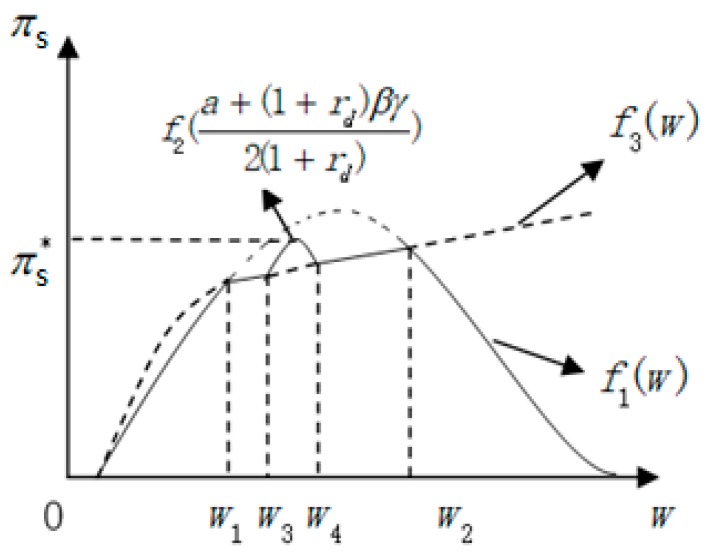
The schematic profit function of the supplier for Case A6.

**Figure A3:**
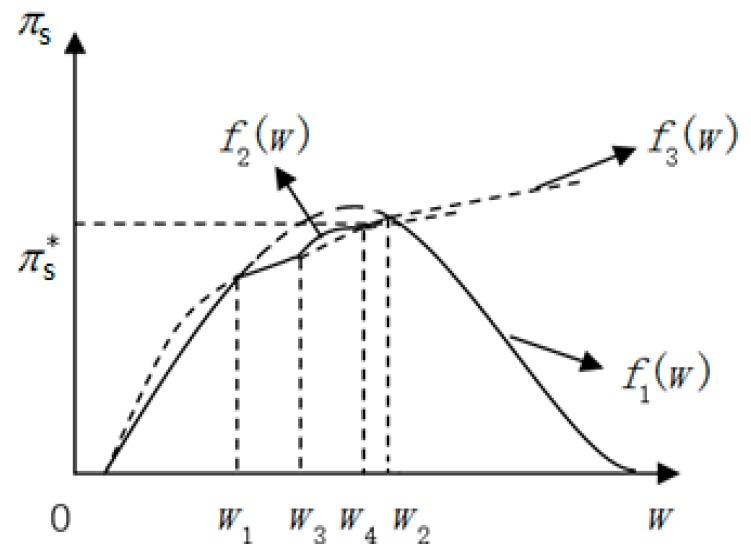
The schematic profit function of the supplier for Case A7.

Note that $\sqrt{a^{2}/{\left(1+r_{d}\right)}^{2}-8bK/(1+r_{d})}<\;\sqrt{a^{2}-8bK}$ holds for all $r_{d}>0$. Then, Lemma 2 explores that there is an interval of the emission tax rates (i.e., $\gamma \in [\sqrt{a^{2}/{\left(1+r_{d}\right)}^{2}-8bK/(1+r_{d})}/\beta ,\;\sqrt{a^{2}-8bK}/\beta )$) such that the supplier’s wholesale price decisions are independent of the regulatory policy. The intuitive reason for this result is that for each of these moderate emission tax rates, the supplier is not willing to choose either a higher wholesale price to transfer the tax cost (βγ) to the retailer or a lower one to increase the retailer’s order quantity (please refer to Figure A3). However, when the tax rate is high enough ($\gamma >\sqrt{a^{2}-8bK}/\beta$), it is profitable for the supplier to take advantage of a high wholesale price to transfer the tax cost to the retailer in spite of a loss of order quantity. Further, for low enough tax rates ($\gamma <\sqrt{a^{2}/{\left(1+r_{d}\right)}^{2}-8bK/(1+r_{d})}/\beta$) with low interest rates ($r_{d}\leq r_{d}^{\#}$), the transferring of the tax cost has a small (negative) effect on the retailer’s order quantity, since the low enough transferred tax cost supports the retailer’s incentive to finance the working capital constraint. Thus the tax cost transferring benefits the supplier again. Finally, as long as the interest rate is high enough ($r_{d}>r_{d}^{\#}$), the retailer’s incentive to finance disappears even if the wholesale price just transfers a low level of the tax cost. This again makes the supplier’s wholesale price decision independent of the regulatory policy represented by γ. In this case (see Figure A2 and Figure A3), although all wholesale prices $w\in [w_{3},w_{4}]$ could induce the retailer to finance a positive amount to relax its working capital constraint, this incentive is suppressed by the supplier’s choice of $w_{2}$($>w_{4}$).

With Lemmas 1 and 2, one can derive Proposition 1 according to the idea of backward induction.

**Proposition** **1.** *The equilibrium decisions and the corresponding profits are as follows:*

(7) $$w^{\#}=\left\{ \begin{array}{l} \frac{a+\beta \gamma }{2},\;\mathrm{if}\;\sqrt{a^{2}-8bK}\leq \beta \gamma \\ \frac{a+(1+r_{d})\beta \gamma }{2(1+r_{d})},\mathrm{if}\;r_{d}\leq r_{d}^{\#},(1+r_{d})\beta \gamma <\sqrt{a^{2}-8bK(1+r_{d})}\;\mathrm{and}\;\sqrt{a^{2}-8bK}>\beta \gamma \\ \frac{a+\sqrt{a^{2}-8bK}}{2},\mathrm{if}\;\left\{r_{d}>r_{d}^{\#}\;\mathrm{or}\;\sqrt{a^{2}-8bK(1+r_{d})}\leq (1+r_{d})\beta \gamma \right\}\;\mathrm{and}\;\beta \gamma <\sqrt{a^{2}-8bK} \end{array}\right.$$

(8) $$q^{\#}=\left\{ \begin{array}{l} \frac{a-\beta \gamma }{4b},\;\mathrm{if}\;\sqrt{a^{2}-8bK}\leq \beta \gamma \\ \frac{a-\left(1+r_{d}\right)\beta \gamma }{4b},\;\mathrm{if}\;r_{d}\leq r_{d}^{\#},(1+r_{d})\beta \gamma <\sqrt{a^{2}-8bK(1+r_{d})}\;(<(1+r_{d})\sqrt{a^{2}-8bK})\\ \frac{2K}{a+\sqrt{a^{2}-8bK}},\;\mathrm{if}\;\left\{r_{d}>r_{d}^{\#}\;\mathrm{or}\;\sqrt{a^{2}-8bK(1+r_{d})}\leq (1+r_{d})\beta \gamma \right\}\;\mathrm{and}\;\beta \gamma <\sqrt{a^{2}-8bK} \end{array}\right.$$

(9) $$L^{\#}=\left\{ \begin{array}{l} \frac{a^{2}-{\left[\left(1+r_{d}\right)\beta \gamma \right]}^{2}}{8b\left(1+r_{d}\right)}-K,\;\mathrm{if}\;r_{d}\leq r_{d}^{\#},(1+r_{d})\beta \gamma <\sqrt{a^{2}-8bK(1+r_{d})}\;\mathrm{and}\;\sqrt{a^{2}-8bK}>\beta \gamma \\ 0,\;\mathrm{otherwise} \end{array}\right.$$

(10) $$\pi_{s}^{\#}=\left\{ \begin{array}{l} \frac{{\left(a-\beta \gamma \right)}^{2}}{8b},\;\mathrm{if}\;\sqrt{a^{2}-8bK}\leq \beta \gamma \\ \frac{{\left[a-\left(1+r_{d}\right)\beta \gamma \right]}^{2}}{8b\left(1+r_{d}\right)},\;\mathrm{if}\;r_{d}\leq r_{d}^{\#},(1+r_{d})\beta \gamma <\sqrt{a^{2}-8bK(1+r_{d})}\;\mathrm{and}\;\sqrt{a^{2}-8bK}>\beta \gamma \\ K\left[1-\frac{2\beta \gamma }{a+\sqrt{a^{2}-8bK}}\right],\;\mathrm{otherwise} \end{array}\right.$$

(11) $$\pi_{r}^{\#}=\left\{ \begin{array}{l} \frac{{\left(a-\beta \gamma \right)}^{2}}{16b},\;\mathrm{if}\;\sqrt{a^{2}-8bK}\leq \beta \gamma \\ \frac{{\left[a-\left(1+r_{d}\right)\beta \gamma \right]}^{2}}{16b}+Kr_{d},\;\mathrm{if}\;r_{d}\leq r_{d}^{\#},(1+r_{d})\beta \gamma <\sqrt{a^{2}-8bK(1+r_{d})}\;\mathrm{and}\;\sqrt{a^{2}-8bK}>\beta \gamma \\ a\frac{2K}{a+\sqrt{a^{2}-8bK}}-b{\left(\frac{2K}{a+\sqrt{a^{2}-8bK}}\right)}^{2}-K,\;\mathrm{otherwise} \end{array}\right.$$

**Figure 1:**
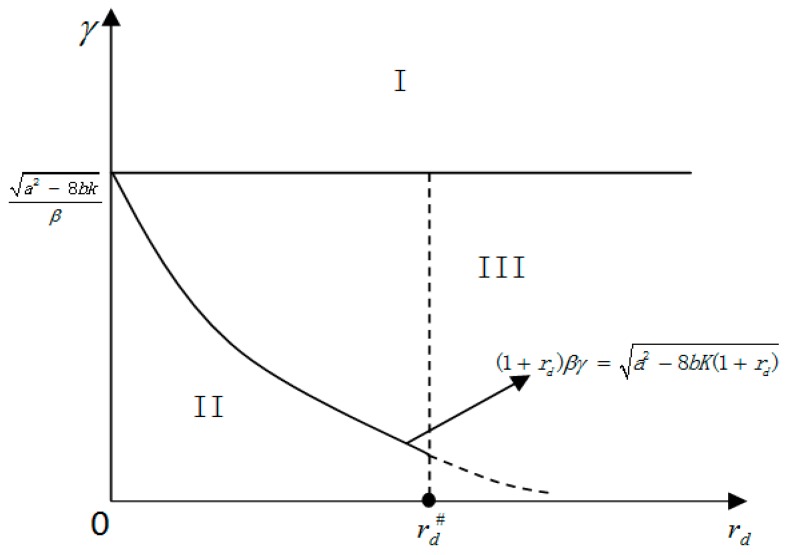
Parameter regions for three different equilibriums.

Based on the equilibrium results in Proposition 1, we identify three regions in the $r_{d}-\gamma$ plane, which are illustrated in Figure 1. Region I corresponds to the case of $\gamma \geq \sqrt{a^{2}-8bK}/\beta$ (or $\sqrt{a^{2}-8bK}\leq \beta \gamma$). Region II represents the case of $\gamma <\sqrt{a^{2}/{\left(1+r_{d}\right)}^{2}-8bK(1+r_{d})}/\beta$ (or $(1+r_{d})\beta \gamma <$ $\sqrt{a^{2}-8bK(1+r_{d})}$) and $r_{d}\leq r_{d}^{\#}$. Region III includes all other parameter pairs (specifically, subregion III-1: $r_{d}>r_{d}^{\#}$ an $\gamma <\sqrt{a^{2}/{\left(1+r_{d}\right)}^{2}-8bK(1+r_{d})}/\beta$, or subregion III-2: $\gamma \in [\sqrt{a^{2}/{\left(1+r_{d}\right)}^{2}-8bK(1+r_{d})}/\beta ,$ $\sqrt{a^{2}-8bK}/\beta )$ for all $r_{d}$).

According to Proposition 1, when the $r_{d}-\gamma$ parameter pairs fall in Region I, where the emission tax rates are very high, the supplier uses a high wholesale price $w^{\#}=(a+\beta \gamma )/2$ to transfer the high tax cost (βγ) to the retailer. In response to the supplier’s high wholesale price, the retailer chooses a low order quantity $q^{\#}=(a-\beta \gamma )/(4b)$ so that its working capital constraint is not binding. Thus there no need for the retailer to finance, implying that financial development has no impact on the operations of the supply chain and thus does not affect GHG emissions at all. Clearly, given that GHG emission is positively associated with the supplier’s production, the response of the retailer’s order quantity (the supplier’s production of the quantity) implies the effectiveness of the regulatory policy to manage GHG emissions in the supply chain.

In Region II, the emission tax rates are low enough (i.e., $\gamma <\sqrt{a^{2}-8bK(1+r_{d})}/(1+r_{d})\beta$). This leads to enough low emission tax costs (βγ) for the supplier. This would in turn allow the supplier to choose low wholesale prices ($w^{\#}=[a+(1+r_{d})\beta \gamma ]/[2(1+r_{d})]$) to transfer these tax costs. If the marginal financing cost is low enough ($r_{d}\leq r_{d}^{\#}$), low wholesale prices leave the retailer with (relatively) high profit margins to cover the marginal financing cost so that it is willing to finance a positive amount from the bank. This tends to enhance the retailer’s order quantity ($q^{\#}=(a-(1+r_{d})\beta \gamma )/(4b)$). This again strengthens the supplier’s incentive to use low wholesale prices (but higher than the low tax costs). In summary, the supplier responds to these low enough tax rates by using the wholesale price to transfer the low enough tax costs, and at the same time, the resulting low tax cost transferring wholesale prices incentivize the retailer to finance. Therefore, when the $r_{d}-\gamma$ parameter pairs are in Region II, the supplier actively responds to the emission regulatory policy (represented by γ) and then induces the retailer to take the financial development level (captured by $r_{d}$) into account when choosing a financing amount. Thus, both emission regulatory policies and financial development affect the supply chain’s operations as well as GHG emissions (given that the supplier’s GHG emissions are positively correlated to its production).

In Region III, we have two subregions. In subregion III-1, for low enough emission tax rates $\gamma <\sqrt{a^{2}/{\left(1+r_{d}\right)}^{2}-8bK(1+r_{d})}/\beta$, if the supplier chooses $w^{\#}=[a+(1+r_{d})\beta \gamma ]/[2(1+r_{d})]$ to transfer the tax costs (βγ), the retailer would be willing to finance a positive amount. However, as indicated by the inequality (A2) in the proof of Lemma 2, this is not optimal for the supplier when $r_{d}>r_{d}^{\#}$, since the high interest rate weakens the retailer’s motivation to finance and then enlarge its order quantity from the supplier. Thus the supplier chooses the highest wholesale price $w^{\#}=w_{2}$ that, on the one hand, suppresses the retailer’s incentive to finance, and on the other hand, enables the supplier to produce the lowest quantity (so as to save the emission tax cost as much as possible) that just exhausts the retailer’s current working capital. That is, the supplier’s revenue is constant at the amount of the retailer’s working capital (K). As a result, any impact of either an emissions regulatory policy or financial development is buffered by the supplier’s wholesale price choices. In subregion III-2, the emission tax rates are relatively high (i.e., $\gamma \geq \sqrt{a^{2}/{\left(1+r_{d}\right)}^{2}-8bK(1+r_{d})}/\beta$). In this subregion, if $r_{d}\leq r_{d}^{\#}$, even if the supplier tries to use relatively high wholesale prices to transfer the relatively high tax costs to the retailer, the relatively high wholesale prices do not give the retailer any incentive to finance ($L^{\#}=0$). This leads to constant revenue for the supplier. If $r_{d}>r_{d}^{\#}$, the supplier is not willing to transfer the tax costs by wholesale prices, nor does the retailer have any incentive to finance. This again results in constant revenue for the supplier and the regulatory policies and financial development to be ineffective at managing GHG emissions in the supply chain. 

To summarize, Proposition 1 identifies conditions (as illustrated in Figure 1) under which regulatory policies and/or financial development level effectively affect GHG emission behaviors in supply chains. More specifically, if the GHG emission tax rate is high enough (Region I), the regulatory policy via taxing is effective, while financial development cannot affect the supply chain’s emissions at all. If the GHG emission tax rate and financial development level are high enough (Region II), both regulatory policies and financial development affect the supply chain’s emissions, implying that to effectively manage emissions, a regulatory policy needs to fit the specific level of financial development. More interestingly, if the GHG emission tax rate and financial development level fall in Region III, all impacts of regulatory policies and financial development are buffered by the supplier’s wholesale decisions, leading to the ineffectiveness of regulatory policies and financial development to influence GHG emissions in the supply chain.

## 5. Comparative Statistics

Now we discuss how exogenous parameters $r_{d}$, γ, and β affect the equilibrium results and the corresponding managerial insights.

**Proposition** **2.** *In Region II, where financial development is able to affect the operations of the supply chain (i.e.,* $r_{d}\leq r_{d}^{\#}$ *and* $(1+r_{d})\beta \gamma <\sqrt{a^{2}-8bK(1+r_{d})}$*), a higher interest rate of bank financing (*$r_{d}$*) leads to a lower wholesale price (*$w^{\#}$*), a lower equilibrium ordering quantity (* $q^{\#}$*), a lower amount of bank financing (*$L^{\#}$*), and a lower level of profitability for the supplier (*$\pi_{s}^{\#}$*). However, the retailer’s profit (* $\pi_{r}^{\#}$*) decreases in* $r_{d}$ *for all* $r_{d}\in \left(0,r_{d}^{a}\right)$ *and increases in* $r_{d}$ *for all* $r_{d}\in \left(r_{d}^{a},r_{d}^{\#}\right)$

Proposition 2 demonstrates that in Region II, where there is an impact from financial development, financial development facilitates the production of the supply chain and enhances the supplier’s profitability. But the bad news is that higher production quantity implies a higher level of GHG emissions. Thus financial development brings a conflict between the incentive of production and the GHG abatement requirement. The reason is as follows. First, a higher level of financial development leads to a lower level of financing cost (the interest rate, in our case) and thus incentivizes the retailer to relax its working capital constraints. Second, the relaxation of working capital constraint increases the retailer’s (derived) demand for the supplier’s product; that is, for each given wholesale price in the case of $w_{3}\leq w\leq w_{4}$ in (2), the retailer is willing to buy more products from the supplier. Third, faced with increased (derived) demand from the retailer and the constant marginal regulation-induced cost (βγ) due to the emission tax regulation, the supplier expands its production and sells more products to the retailer at a higher wholesale price. Finally, note that the supplier’s GHG emissions increase in its output, then a higher level of GHG emissions follows accordingly from the supplier’s production expansion. This explores a path along which financial development positively affects the GHG emissions of the producing firm in a supply chain (the supplier, in our case) and thus provides a strategic base to support the positive part of the inverted U-shaped relationship, observed in [14,15,16,17], between GHG emissions and financial development. However, as we will see in Propositions 3 and 4, a strengthened regulatory policy (respectively, a bigger γ) or an improved GHG abatement technology (respectively, a smaller β) makes the supplier decrease its GHG emissions. Therefore, this inverted U-shaped relationship, in our setting, can be attributed to a comparison between these two countereffects. In settings where the levels of financial development, regulatory policies, and GHG abatement technologies can change simultaneously, the positive (negative) part can be observed when the effect of financial development dominates (is dominated by) the effect of a strengthened regulatory policy and/or an improved GHG abatement technology.

Proposition 2 also reveals a seemingly counterintuitive result: the interest rate of financing cost as a U-shape impacts the retailer’s profit, even if the financing cost (i.e., the interest rate) decreases in the level of financial development. The reason is as follows. Note that the retailer’s equilibrium profit consists of two parts (see [11]). The first term represents the profit from the product transaction (buying from the supplier and reselling to final consumers), and the second term represents the profit from the opportunity revenue of the retailer’s currently owned working capital without any external financing (i.e., saving the retailer’s financing cost). For a high (low) enough interest rate ($r_{d}\geq (<)r_{d}^{a}$), the negative impact of an increase in the interest rate on the retailer’s product-transaction profit is dominated by (dominates) the positive effect on the opportunity revenue. This leads to a U-shaped impact of the interest rate on the retailer’s profitability.

**Proposition** **3.** *(a) In Region I (i.e.,* $\beta \gamma >\sqrt{a^{2}-8bK}$*), a higher GHG emission tax rate* γ *leads to a higher wholesale price (*$w^{\#}$*), a lower ordering quantity (* $q^{\#}$*), and a lower level of profitability for the supplier (*$\pi_{s}^{\#}$*) and the retailer (*$\pi_{r}^{\#}$*), but it has no impact on the amount of bank financing (*$L^{\#}$*); (b) in Region II (i.e.,* $r_{d}\leq r_{d}^{\#}$ *and* $(1+r_{d})\beta \gamma <\sqrt{a^{2}-8bK(1+r_{d})}$*), a higher GHG emission tax rate* γ *leads to a higher wholesale price (*$w^{\#}$*), a lower ordering quantity (*$q^{\#}$*), a lower amount of bank financing (*$L^{\#}$*), and a lower level of profitability for the supplier (*$\pi_{s}^{\#}$*) and the retailer (*$\pi_{r}^{\#}$*); (c) in Region III, it does not have any impact on the wholesale price (*$w^{\#}$*), the ordering quantity (*$q^{\#}$*), the amount of bank financing (*$L^{\#}$*), or the retailer’s profit (*$\pi_{r}^{\#}$*), but it results in a lower level of profitability for the supplier (*$\pi_{s}^{\#}$*).*

Proposition 3 explores that in Regions I and II, an increase in the GHG emission tax rate raises the supplier’s marginal cost and thus reduces its producing quantity and drives up the wholesale price at which the product is sold to the retailer. Naturally, this leads to a lower level of profitability for both supply chain members. Further, the positive association between the supplier’s production and GHG emissions implies that the supplier’s GHG emissions decrease as the regulation is strengthened by a higher GHG emission tax rate. However, in Region III, since the supplier always suppresses the retailer’s financing motivation by choosing the highest wholesale price to exhaust the retailer’s (fixed) working capital, its revenue is exactly fixed at the amount of the retailer’s working capital. Thus, a higher GHG emission tax rate increases the supplier’s cost and thus leads to a lower profit.

Together with Proposition 2, Proposition 3 provides two important managerial insights. First, Proposition 2 and Proposition 3b show that when financial development and GHG emission regulatory policies based on emission taxes can affect the supplier’s production (in Region II), whether a stricter regulatory policy in terms of a higher emission tax rate leads to lower GHG emissions depends on the level of financial development. Indeed, a higher level of financial development weakens the emission-reducing effect of a stricter regulatory policy. Thus, to realize a given GHG emission objective, a regulator has to seriously incorporate financial development into the framework of regulatory policy decisions. Second, Proposition 3c highlights that in Region III, where regulatory policies cannot work to affect the supplier’s production, any change in regulatory policies just lowers the supplier’s profit without any effect on GHG emissions. The implication is that if Region III is present, it is difficult to justify a stricter GHG regulatory policy from the GHG emission perspective. It would be interpreted as a trick of the regulator to snatch firms’ business benefits. Thus it is better for the regulator to loosen its regulatory policy by choosing an emission tax rate on the lower boundary of Region III.

**Proposition** **4.** *(a) In Region I (i.e.,* $\beta \gamma >\sqrt{a^{2}-8bK}$*), a higher GHG emission rate of the supplier production* β *leads to a higher wholesale price (* $w^{\#}$*), a lower ordering quantity (*$q^{\#}$*), and a lower level of profitability for the supplier (*$\pi_{s}^{\#}$*) and the retailer (*$\pi_{r}^{\#}$*), but it has no impact on the amount of bank financing (*$L^{\#}$*); (b) in Region II (i.e.,* $r_{d}\leq r_{d}^{\#}$ *and* $(1+r_{d})\beta \gamma <\sqrt{a^{2}-8bK(1+r_{d})}$*), a higher GHG emission rate of the supplier production* β *leads to a higher wholesale price (* $w^{\#}$*), a lower ordering quantity (*$q^{\#}$*), a lower amount of bank financing (*$L^{\#}$*), and a lower level of profitability for the supplier (*$\pi_{s}^{\#}$*) and the retailer (*$\pi_{r}^{\#}$*); (c) in Region III, it does not have any impact on the wholesale price (*$w^{\#}$*), the ordering quantity (*$q^{\#}$*), the amount of bank financing (*$L^{\#}$*), or the retailer’s profit (*$\pi_{r}^{\#}$*), but it results in a lower level of profitability for the supplier (*$\pi_{s}^{\#}$*).*

Note that the results in Proposition 4 mainly depend on the assumption of γ>0. Given this assumption, Proposition 4 reveals that a lower β leads to a win-win outcome, i.e., higher profitability for both supplier and retailer (except that the retailer’s profit stays constant). This implies that GHG regulations are necessary for profit-pursuing supply chain members to lower the unit GHG emissions accompanying the production in a supply chain (whenever possible). From the viewpoint of supply chain members, when faced with GHG emission regulations, there is an opportunity for them to invest in some GHG abatement technologies to raise their economic performance in terms of profitability. Finally, the win-win outcome further implies that supply chain members are willing to collaborate on developing and adopting some advanced GHG abatement technologies.

## 6. Conclusions

To understand the impacts of GHG emission regulations and the level of financial development on firms’ GHG emissions from a micro perspective, we build a two-echelon supply chain model in which the upstream supplier’s production is accompanied by GHG emissions and the downstream retailer is faced with a working capital constraint. GHG emissions are taxed by a regulator, while the working capital constraint can be relaxed by the retailer’s financing from an external bank. With the subgame perfect equilibrium, we show that (1) there is a set of GHG emission tax rates such that the supplier’s GHG emission keeps unchanged; (2) if the GHG emission tax rate is high enough, its increase reduces the supplier’s GHG emissions, while this is independent of any financial development level; (3) if both the GHG emission tax rate and the level of financial development are high enough, the tax rate has a negative impact on the supplier’s emissions, while the impact of financial development is positive; (4) financial development does not have any impact on the supplier’s GHG emissions if they are at a low enough level; and (5) both the supplier’s and the retailer’s profits decrease with the GHG emission tax rate and the GHG emissions per unit of the supplier’s production, while the financial development, if it is at a high enough level, positively affects both supply chain members’ profitability. 

The policy implications of these results are as follows. First, to evaluate the effectiveness of an emission tax policy to regulate firms’ GHG emissions in a supply chain, policy-makers have to seriously take into account the level of financial development. The reason is that the corresponding financing cost affects a resource-constrained node firm’s financing decisions and production operations in the supply chain. In our model setting, the retailer’s decision on whether to finance its working capital is the main determinant of whether an emission tax policy effectively induces the supplier’s production and GHG emissions. Second, although market-based environmental regulations (MERs) are useful to incentivize a firm’s GHG emission abatement [50], our results point out that an emission tax, as the MER instrument, may not work. More precisely, these ineffective scenarios correspond to Region III in Figure 1. This implies that when the emission tax instrument fails to work, the policy-maker may resort to some command-and-control regulation, such as specifying appropriate emission caps. Third, our results reveal that the only impact of any change in the tax rate in the ineffective region is to reallocate the profit of the GHG-emitting firm (the supplier, in our model). This may trigger additional conflict between the regulator and the regulated firm. Finally, our results also highlight that when financial development is at a high enough level, further promotion of it is two-edged sword. It enhances firms’ profitability at the cost of environmental deterioration due to heightened GHG emissions. However, this economic profitability enhancement may potentially support firms’ investing in GHG abatement technologies. Such investments are expected to decrease GHG emissions, according to the Porter hypothesis. Thus, from a long-run viewpoint, promoting financial development would achieve a win-win outcome if the increase in firms’ short-run profitability were converted into long-run investments in GHG abatement technologies.

A limitation of the present research is that we exclude the possibility of trade credits (as a financing venue) with the supply chain. Especially when the retailer’s financing from an external bank is impossible or has restrictive cost, is there any motivation for the supplier to finance the retailer with some trade credits (e.g., receivables)? In the case of trade credits, how does a GHG emission regulation affect the supply chain operations (and the corresponding emissions)? These questions are worth addressing in the future. Second, our results imply some profitability potential from reducing the unit emission rate of the supplier’s production. Thus another valuable extension is to explicitly incorporate financial development into the model by assuming that the supplier is resource-constrained and there are opportunities to invest in some abatement technologies to reduce its GHG emission rate. On the other hand, if the retailer’s resources are abundant, there is a possibility for both supply chain members to collaborate on such abatement technologies. In this case, how does financial development affect their collaboration? Third, we assume that the regulatory policy and the interest rate are exogenous. However, in reality, both a regulator and a bank, to a certain degree, have some influence on choosing a regulatory policy and deciding the interest rate, respectively. This calls for extensions of our model to capture the regulator’s and the bank’s strategic behaviors. Although these extensions will inevitably increase the difficulty of solving the model, they are expected to bring more precise understanding of the real-world interactions among supply chain members, regulators, and banks. 

## Figures and Tables

**Figure A1:**
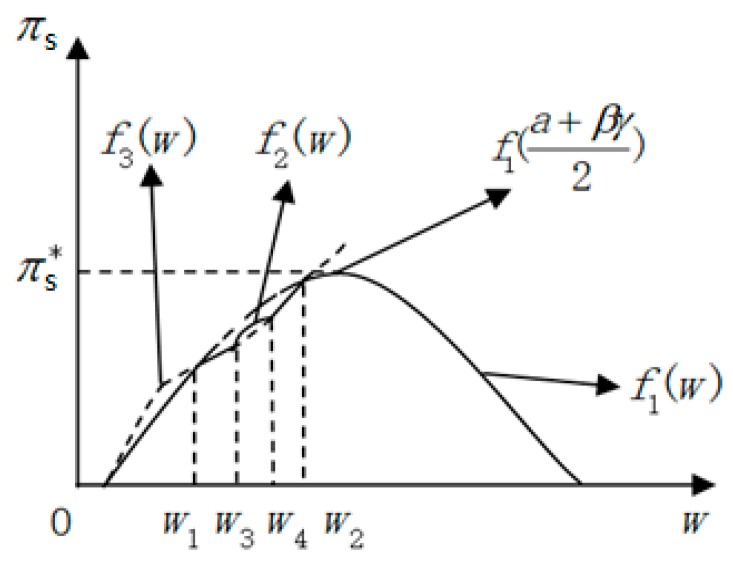
The schematic profit function of the supplier for Case A5.

## Appendix A. Proofs of Lemmas and Propositions

**Proof** **of** **Lemma** **1.** The Lagrangian function for the maximization problem (1) is

$$\Phi (L,q,\lambda_{1},\lambda_{2})=\pi_{r}\left(L,q\right)+\lambda_{1}\left(K+L-wq\right)+\lambda_{2}L$$

where $\lambda_{1}$ and $\lambda_{2}$ are the Lagrange multipliers for the constraints.

By the Kuhn-Tucker theorem, the first-order conditions are

(A1) $$\begin{array}{ll} \frac{\partial \Phi }{\partial q}=a-2bq-w-\lambda_{1}w=0 & \left(a\right)\\ \frac{\partial \Phi }{\partial L}=-r_{d}+\lambda_{1}+\lambda_{2}=0 & \left(b\right)\\ \lambda_{1}\left(K+L-wq\right)=0 & \left(c\right)\\ \lambda_{2}L=0 & \left(d\right)\\ \lambda_{1}\geq 0,\lambda_{2}\geq 0 & \left(e\right) \end{array}$$

We discuss below the optimal solution in a case-by-case manner.

**Case A1:** $\lambda_{1}=0$ and $\lambda_{2}=0$. We have ∂Φ/∂L<0. But $\lambda_{2}=0$ implies L>0. This is a contradiction.

**Case A2:** $\lambda_{1}>0$ and $\lambda_{2}=0$. From the second condition in A1b, we have $\lambda_{1}=r_{d}$. Further, the second condition in A1b implies $q=[a-(1+r_{d})w]/(2b)$. Thus $\lambda_{2}=0$ implies that for $q(w)=[a-(1+r_{d})w]/(2b)$ to be the optimal ordering quantity, it must be that $w\in (w_{3},w_{4})$. Finally, $\lambda_{1}=r_{d}>0$ implies that the optimal amount of bank financing is $L(w)=\frac{[a-(1+r_{d})w]w}{2b}-K\geq 0$.

**Case A3:** $\lambda_{1}=0$ and $\lambda_{2}>0$. It follows from $\lambda_{2}>0$ that the optimal amount of bank financing is L(w)=0. Then by $\lambda_{1}=0$, the optimal ordering quantity is q(w)=(a−w)/(2b). However, for q(w)=(a−w)/(2b) to be optimal, $\lambda_{1}=0$ requires $w\notin [w_{1},w_{2}]$.

**Case A4:** $\lambda_{1}>0$ and $\lambda_{2}>0$. It immediately follows that L(w)=0 and wq(w)=K⇔q(w)=K/w. Further, from the first and the second conditions in A1b, we have

$$\lambda_{1}=(a-w-2bK/w)/w>0\Leftrightarrow w\in (w_{1},w_{2})$$

$$\lambda_{2}=r_{d}-\lambda_{1}>0\Leftrightarrow w\notin [w_{3},w_{4}]$$

Lemma 1 is thus proved by summarizing the above four cases.

**Proof** **of** **Lemma** **2.** We first define

$$f_{1}(w)=\frac{\left(w-\beta \gamma )(a-w\right)}{2b},f_{2}(w)=\frac{\left(w-\beta \gamma )[a-(1+r_{d})w\right]}{2b}$$

$$f_{3}(w)=\frac{K(w-\beta \gamma )}{w},w_{0}=\frac{a+(1+r_{d})\beta \gamma }{2(1+r_{d})}$$

Below, we derive the supplier’s optimal wholesale prices, respectively, for the three cases specified in the profit function (4).

**Case** **A5:** $\sqrt{a^{2}-8bK}\leq \beta \gamma \Leftrightarrow (a+\beta \gamma )/2\geq w_{2}$ (please refer to Figure A1). Note that w=(a+βγ)/2 is the global maximizer of $f_{1}(w)$. Therefore, for $w>w_{2}$ and w≠(a+βγ)/2, we have $\pi_{s}((a+\beta \gamma )/2)>f_{1}(w)=\pi_{s}(w)$. Similarly, for all $w<w_{1}(<(a+\beta \gamma )/2)$, we have $\pi_{s}((a+\beta \gamma )/2)>f_{1}(w)=\pi_{s}(w)$.

For all $w\in [w_{3},w_{4}]$, we have $\pi_{s}(w)=f_{2}(w)<f_{1}(w)\leq f_{1}(w_{4})<\pi_{s}((a+\beta \gamma )/2)$. Note that $\pi_{s}(w)=f_{3}(w)$ is increasing in w. Thus we have $\pi_{s}(w)=f_{3}(w)\leq f_{1}(w_{2})<\pi_{s}((a+\beta \gamma )/2)$. In summary, we obtain that when $(a+\beta \gamma )/2\geq w_{2}$, the optimal wholesale price is $w^{\#}=(a+\beta \gamma )/2$.

**Case** **A6:** $r_{d}\leq r_{d}^{\#},(1+r_{d})\beta \gamma <\sqrt{a^{2}-8bK(1+r_{d})}$ and $\sqrt{a^{2}-8bK}>\beta \gamma$ (please refer to Figure A2). In this case, we have $(a+\beta \gamma )/2<w_{2}$ and $w_{0}=[a+(1+r_{d})\beta \gamma ]/[2(1+r_{d})]<w_{4}$. Note that for all $w>w_{2}(>(a+\beta \gamma )/2)$, $\pi_{s}(w)=f_{1}(w)$ is decreasing in w. Then $\pi_{s}(w)=f_{1}(w)<f_{1}(w_{2})=\pi_{s}(w_{2})$. Similarly, for all $w\leq w_{1}(<(a+\beta \gamma )/\beta \gamma )$, we have $\pi_{s}(w)=f_{1}(w)<f_{1}(w_{1})$. Further, because $w_{1}<w_{2}$ and $f_{3}(w)$ increase in w, we have

$$\pi_{s}(w)=f_{1}(w)<f_{1}(w_{1})=\underset{w\to w_{1}^{+}}{\lim}f_{3}(w)<\underset{w\to w_{2}^{-}}{\lim}f_{3}(w)=f_{1}(w_{2})=\pi_{s}(w_{2})$$

For all $w_{1}<w<w_{3}$ or $w_{4}<w<w_{2}$, the monotonicity of function $f_{3}(w)$ and the continuity of $\pi_{s}(w)$ imply that

$$\pi_{s}(w)<\underset{w\to w_{2}^{-}}{\lim}f_{3}(w)=f_{1}(w_{2})=\pi_{s}(w_{2})$$

For $w_{3}\leq w\leq w_{4}$, due to the concavity of $\pi_{s}(w)=f_{2}(w)$, $w_{0}=[a+(1+r_{d})\beta \gamma ]/[2(1+r_{d})]$ is a local maximum point. Thus, $w_{0}=[a+(1+r_{d})\beta \gamma ]/[2(1+r_{d})]$ and $w_{2}=(a+\sqrt{a^{2}-8bK})/2$ are also two local maximum points with profits $\pi_{s}(w_{0})$ and $\pi_{s}(w_{2})$, respectively.

Note that

$$\pi_{s}(w_{0})\geq \pi_{s}(w_{2})\Leftrightarrow H(r_{d})=\frac{{\left[a-(1+r_{d})\beta \gamma \right]}^{2}}{8b(1+r_{d})}-K\left(1-\frac{2\beta \gamma }{a+\sqrt{a^{2}-8bK}}\right)\geq 0$$

Obviously, for all $r_{d}\in [0,a/\beta \gamma -1]$, $H^{\prime }(r_{d})<0$. Further,

$$\begin{array}{ll} H(0) & =\frac{{\left[a-\beta \gamma \right]}^{2}}{8b}-K\left(1-\frac{2\beta \gamma }{a+\sqrt{a^{2}-8bK}}\right)>\frac{{\left(a-\beta \gamma \right)}^{2}}{8b}-K\left(1-\frac{2\beta \gamma }{a+\beta \gamma }\right)\\ & =\frac{\left(a-\beta \gamma )(a^{2}-\beta^{2}\gamma^{2}-8bK\right)}{8b(a+\beta \gamma )}>0 \end{array}$$

(A2) $$\begin{array}{ll} H(r_{d}^{\prime })<0 & \Leftrightarrow a^{2}+{\left(1+r_{d}^{\prime }\right)}^{2}{\left(\beta \gamma \right)}^{2}<8bK(1+r_{d}^{\prime })+2\beta \gamma (1+r_{d}^{\prime })\sqrt{a^{2}-8bK}\\ & \Leftrightarrow 2{\left(1+r_{d}^{\prime }\right)}^{2}{\left(\beta \gamma \right)}^{2}<2\beta \gamma (1+r_{d}^{\prime })\sqrt{a^{2}-8bK}\\ & \Leftrightarrow 2\beta \gamma (1+r_{d}^{\prime })[(1+r_{d}^{\prime })\beta \gamma -\sqrt{a^{2}-8bK}]<0\\ & \Leftrightarrow 2\beta \gamma (1+r_{d}^{\prime })[\sqrt{a^{2}-8bK(1+r_{d}^{\prime })}-\sqrt{a^{2}-8bK}]<0 \end{array}$$

where $r_{d}^{\prime }$ is the unique root of $(1+r_{d})\beta \gamma =\sqrt{a^{2}-8bK(1+r_{d})}$.

Thus, there exists $r_{d}^{\#}\in (0,r_{d}^{\prime })$ such that $\pi_{s}(w_{0})\geq (<)\pi_{s}(w_{2})$ for all $r_{d}\leq (>)r_{d}^{\#}$. As a result, the supplier’s optimal wholesale price is $w^{\#}=w_{0}=[a+(1+r_{d})\beta \gamma ]/[2(1+r_{d})]$ when $r_{d}\leq r_{d}^{\#}$, $\sqrt{a^{2}-8bK}>\beta \gamma$, and $(1+r_{d})\beta \gamma <\sqrt{a^{2}-8bK(1+r_{d})}$.

**Case** **A7:** $(a+\beta \gamma )/2<w_{2}$ and $[a+(1+r_{d})\beta \gamma ]/[2(1+r_{d})]>w_{4}$ (please refer to Figure A3). In this case, we have $(a+\beta \gamma )/2<w_{2}$ and $w_{0}=[a+(1+r_{d})\beta \gamma ]/[2(1+r_{d})]>w_{4}$. Therefore, $\pi_{s}(w)=f_{1}(w)<f_{1}(w_{2})=\pi_{s}(w_{2})$ for all $w>w_{2}(>(a+\beta \gamma )/2)$. In a similar manner, it can be easily checked that for all $w\leq w_{1}(<(a+\beta \gamma )/2)$, $\pi_{s}(w)=f_{1}(w)<f_{1}(w_{1})$. Furthermore, with $w_{1}<w_{2}$, the monotonicity of $f_{3}(w)$, and the continuity of $\pi_{s}(w)$, we have

$$\pi_{s}(w)=f_{1}(w)<f_{1}(w_{1})=\underset{w\to w_{1}^{+}}{\lim}f_{3}(w)<\underset{w\to w_{2}^{-}}{\lim}f_{3}(w)=f_{1}(w_{2})=\pi_{s}(w_{2})$$

Thus, for all $w_{1}<w<w_{3}$ and $w_{4}<w<w_{2}$, the monotonicity of $f_{3}(w)$ and the continuity of $\pi_{s}(w)$ imply that $\pi_{s}(w)<\underset{w\to w_{2}^{-}}{\lim}f_{3}(w)=f_{1}(w_{2})=\pi_{s}(w_{2})$. And for all $w_{3}<w<w_{4}$, both $w_{0}>w_{4}$, the monotonicity of $f_{2}(w)$, and the continuity of $\pi_{s}(w)$ show that $\pi_{s}(w_{3})=f_{3}(w_{3})=\underset{w\to w_{3}^{+}}{\lim}f_{2}(w)<\underset{w\to w_{4}^{-}}{\lim}f_{2}(w)=f_{3}(w_{4})=\pi_{s}(w_{4})$.

In summary, when $(a+\beta \gamma )/2<w_{2}$ and $w_{0}=[a+(1+r_{d})\beta \gamma ]/[2(1+r_{d})]>w_{4}$, the optimal wholesale price is $w^{\#}=w_{2}=(a+\sqrt{a^{2}-8bK})/2$. ☐

**Proof** **of** **Proposition** **2.** We prove this proposition by directly differentiating these equilibrium variables with respect to $r_{d}$ The results are as follows:

$$\frac{\partial w^{\#}}{\partial r_{d}}=-\frac{a}{2}\frac{1}{{\left(1+r_{d}\right)}^{2}}<0,\frac{\partial q^{\#}}{\partial r_{d}}=-\frac{\beta \gamma }{4b}<0$$

$$\frac{\partial L^{\#}}{\partial r_{d}}=-\frac{a^{2}}{8b}\frac{1}{{\left(1+r_{d}\right)}^{2}}-\frac{{\left(\beta \gamma \right)}^{2}}{8b}<0,\;\frac{\partial \pi_{s}^{\#}}{\partial r_{d}}=-\frac{a^{2}-{\left(\beta \gamma \right)}^{2}{\left(1+r_{d}\right)}^{2}}{8b{\left(1+r_{d}\right)}^{2}}<0$$

$$\frac{\partial \pi_{r}^{\#}}{\partial r_{d}}=-\frac{a\beta \gamma -{\left(\beta \gamma \right)}^{2}\left(1+r_{d}\right)}{8b}+K\left\{ \begin{array}{l} <0,\;\mathrm{if}\;r_{d}<r_{d}^{a}\;\\ \geq 0,\;\mathrm{otherwise} \end{array}\right.$$

where $r_{d}^{a}=(a\beta \gamma -\beta^{2}\gamma^{2}-8bK)/{\left(\beta \gamma \right)}^{2}$.

**Proof** **of** **Proposition** **3.** We prove this proposition by directly differentiating all the equilibrium variables with respect to γ The results are as follows:

$$\frac{\partial w^{\#}}{\partial \gamma }=\left\{ \begin{array}{ll} \frac{\beta }{2}>0 & \left(\mathrm{Region}\;I\right)\\ \frac{\beta }{2}>0 & \left(\mathrm{Region}\;\mathrm{II}\right)\\ =0 & \left(\mathrm{Region}\;\mathrm{III}\right) \end{array}\right.\;\frac{\partial q^{\#}}{\partial \gamma }=\left\{ \begin{array}{ll} -\frac{\beta }{4b}<0 & \left(\mathrm{Region}\;I\right)\\ -\frac{(1+r_{d})\beta }{4b}<0 & \left(\mathrm{Region}\;\mathrm{II}\right)\\ =0 & \left(\mathrm{Region}\;\mathrm{III}\right) \end{array}\right.\;\frac{\partial L^{\#}}{\partial \gamma }=\left\{ \begin{array}{ll} =0 & \left(\mathrm{Region}\;I\right)\\ -\frac{(1+r_{d})\beta^{2}\gamma }{4b}<0 & \left(\mathrm{Region}\;\mathrm{II}\right)\\ =0 & \left(\mathrm{Region}\;\mathrm{III}\right) \end{array}\right.\;\frac{\partial \pi_{s}^{\#}}{\partial \gamma }=\left\{ \begin{array}{ll} -\frac{(a-\beta \gamma )\beta }{4b}<0 & \left(\mathrm{Region}\;I\right)\\ -\frac{a-(1+r_{d})\beta \gamma }{4b}\beta <0 & \left(\mathrm{Region}\;\mathrm{II}\right)\\ -\frac{2\beta }{a+\sqrt{a^{2}-8bK}}<0 & \left(\mathrm{Region}\;\mathrm{III}\right) \end{array}\right.\;\frac{\partial \pi_{r}^{\#}}{\partial \gamma }=\left\{ \begin{array}{ll} -\frac{a-\beta \gamma }{8b}\beta <0 & \left(\mathrm{Region}\;I\right)\\ -\frac{a-(1+r_{d})\beta \gamma }{8b}(1+r_{d})\beta <0 & \left(\mathrm{Region}\;\mathrm{II}\right)\\ =0 & \left(\mathrm{Region}\;\mathrm{III}\right) \end{array}\right.$$

**Proof** **of** **Proposition** **4.** Proof of Proposition 4. Similar to the proof of Proposition 3, we differentiate all the equilibrium variables with regard to β as follows:

$$\frac{\partial w^{\#}}{\partial \beta }=\left\{ \begin{array}{ll} \frac{\gamma }{2}>0 & \left(\mathrm{Region}\;I\right)\\ \frac{\gamma }{2}>0 & \left(\mathrm{Region}\;\mathrm{II}\right)\\ =0 & \left(\mathrm{Region}\;\mathrm{III}\right) \end{array}\right.\;\frac{\partial q^{\#}}{\partial \beta }=\left\{ \begin{array}{ll} -\frac{\gamma }{4b}<0 & \left(\mathrm{Region}\;I\right)\\ -\frac{(1+r_{d})\gamma }{4b}<0 & \left(\mathrm{Region}\;\mathrm{II}\right)\\ =0 & \left(\mathrm{Region}\;\mathrm{III}\right) \end{array}\right.\;\frac{\partial L^{\#}}{\partial \beta }=\left\{ \begin{array}{ll} =0 & \left(\mathrm{Region}\;I\right)\\ -\frac{(1+r_{d})\gamma^{2}\beta }{4b}<0 & \left(\mathrm{Region}\;\mathrm{II}\right)\\ =0 & \left(\mathrm{Region}\;\mathrm{III}\right) \end{array}\right.\;\frac{\partial \pi_{s}^{\#}}{\partial \beta }=\left\{ \begin{array}{ll} -\frac{(a-\beta \gamma )\gamma }{4b}<0 & \left(\mathrm{Region}\;I\right)\\ -\frac{a-(1+r_{d})\beta \gamma }{4b}\gamma <0 & \left(\mathrm{Region}\;\mathrm{II}\right)\\ -\frac{2\gamma }{a+\sqrt{a^{2}-8bK}}<0 & \left(\mathrm{Region}\;\mathrm{III}\right) \end{array}\right.\;\frac{\partial \pi_{r}^{\#}}{\partial \beta }=\left\{ \begin{array}{ll} -\frac{a-\beta \gamma }{8b}\gamma <0 & \left(\mathrm{Region}\;I\right)\\ -\frac{a-(1+r_{d})\beta \gamma }{8b}(1+r_{d})\gamma <0 & \left(\mathrm{Region}\;\mathrm{II}\right)\\ =0 & \left(\mathrm{Region}\;\mathrm{III}\right) \end{array}\right.$$
